# Supplementary material for: Bracket Coding: An Emergent Balance Between Temporal Integration and Segregation in Early Visual Population Activity
Source: bioRxiv. 2026 Jul 22:2026.05.31.729124. Preprint. [Version 3] doi: 10.64898/2026.05.31.729124 (PMC13252203; doi:10.64898/2026.05.31.729124)
Supplement: Supplement 1 [file NIHPP2026.05.31.729124v3-supplement-1.pdf]

bioRxiv preprint doi: https://doi.org/10.64898/2026.05.31.729124; this version posted July 22, 2026. The copyright holder for this preprint (which was not certified by peer review) is the author/funder, who has granted bioRxiv a license to display the preprint in perpetuity. It is made available under a CC-BY 4.0 International license.

Supplementary Figures

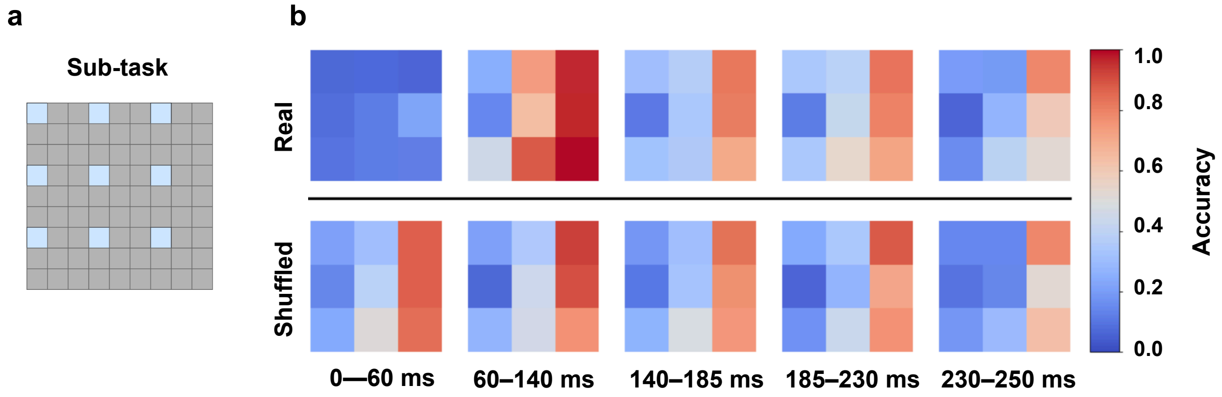

Supplementary Figure S1: **Class-specific decoding profiles across extracted brackets in a representative Gabor subtask.** **(a)** Schematic of the representative subtask used for this analysis. **(b)** Heat maps showing the decoding accuracy profiles of these selected classes across brackets extracted from the real population activity. Each heat map summarizes class-wise decoding performance when the decoder was provided only with spike information integrated within a single bracket. The color in each heat map indicates the fraction of correctly classified trials for each class within that bracket. In the real data, the decoding profile of individual classes changes substantially from one bracket to another, indicating that the decodability of a given class evolves over time. In the shuffled data, although some classes remain easier to decode than others, the temporal changes in class-wise decoding profiles are more uniform.

bioRxiv preprint doi: https://doi.org/10.64898/2026.05.31.729124; this version posted July 22, 2026. The copyright holder for this preprint (which was not certified by peer review) is the author/funder, who has granted bioRxiv a license to display the preprint in perpetuity. It is made available under a CC-BY 4.0 International license.

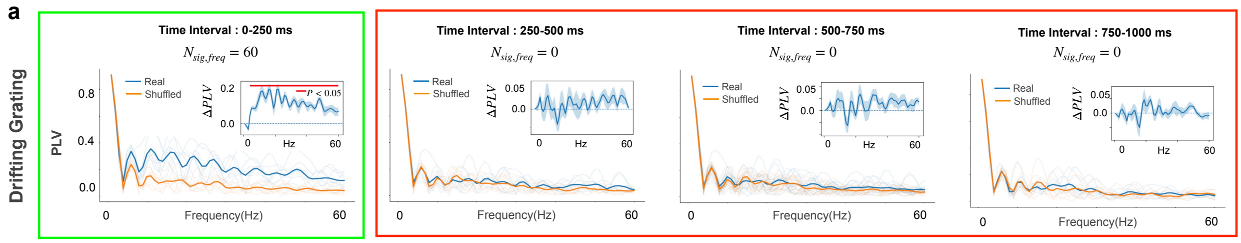

Supplementary Figure S2: **Bracket coding is restricted to early stimulus-evoked response in drifting gratings. (a)** Sweeping–integration analysis of drifting-grating responses performed separately in consecutive 250ms windows across the first second of stimulus presentation. For each interval, phase-locking values (PLVs) between trough times and sinusoids of different frequencies are shown for the real data (blue) and temporally shuffled surrogates (orange), together with the corresponding ΔPLV spectra (insets). Clear bracket structure was observed only in the first 0–250ms interval, which showed a larger PLV in the real data than in the shuffled data and a nonzero number of significant frequencies ($N_{\text{sig,freq}} = 60$), whereas the subsequent intervals (250–500, 500–750, and 750–1000 ms) showed no significant frequencies ($N_{\text{sig,freq}} = 0$).

bioRxiv preprint doi: https://doi.org/10.64898/2026.05.31.729124; this version posted July 22, 2026. The copyright holder for this preprint (which was not certified by peer review) is the author/funder, who has granted bioRxiv a license to display the preprint in perpetuity. It is made available under a CC-BY 4.0 International license.

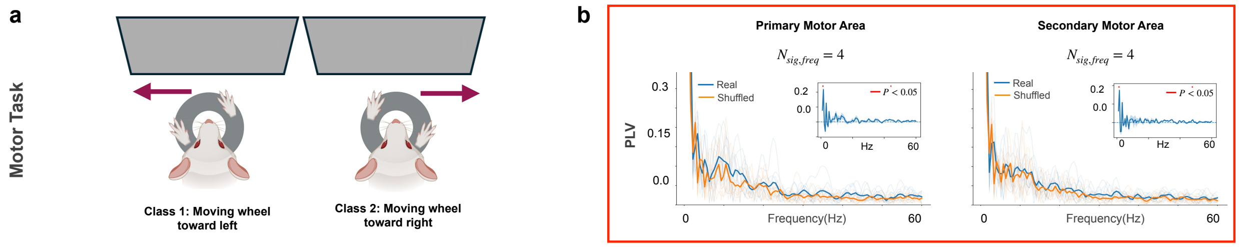

Supplementary Figure S3: **Bracket coding of movement is present but weak in motor regions. (a)** Schematic of motor choice decoding in the IBL dataset. Neural activity from motor regions was used to decode the animal's upcoming behavioral choice, defined as wheel movement toward the left or toward the right. The analysis was performed on the 400 ms period immediately preceding movement onset. Two subtasks were defined by independently splitting left and right movement trials into two equal halves. **(b)** PLV spectra for neuronal populations in primary motor area (MOp) and secondary motor area (MOs), shown for real data (blue) and temporally shuffled surrogates (orange), with corresponding ΔPLV spectra in the insets. In contrast to visual regions, motor populations exhibited weak bracket structure, with only a small number of significant frequencies in both MOp and MOs ($N_{\mathrm{sig,freq}} = 4$). These results suggest that bracket coding is not a general feature of all task-engaged populations, but is strongest in sensory responses that are tightly aligned to stimulus onset and is substantially weaker in motor-related activity associated with action execution.

bioRxiv preprint doi: https://doi.org/10.64898/2026.05.31.729124; this version posted July 22, 2026. The copyright holder for this preprint (which was not certified by peer review) is the author/funder, who has granted bioRxiv a license to display the preprint in perpetuity. It is made available under a CC-BY 4.0 International license.

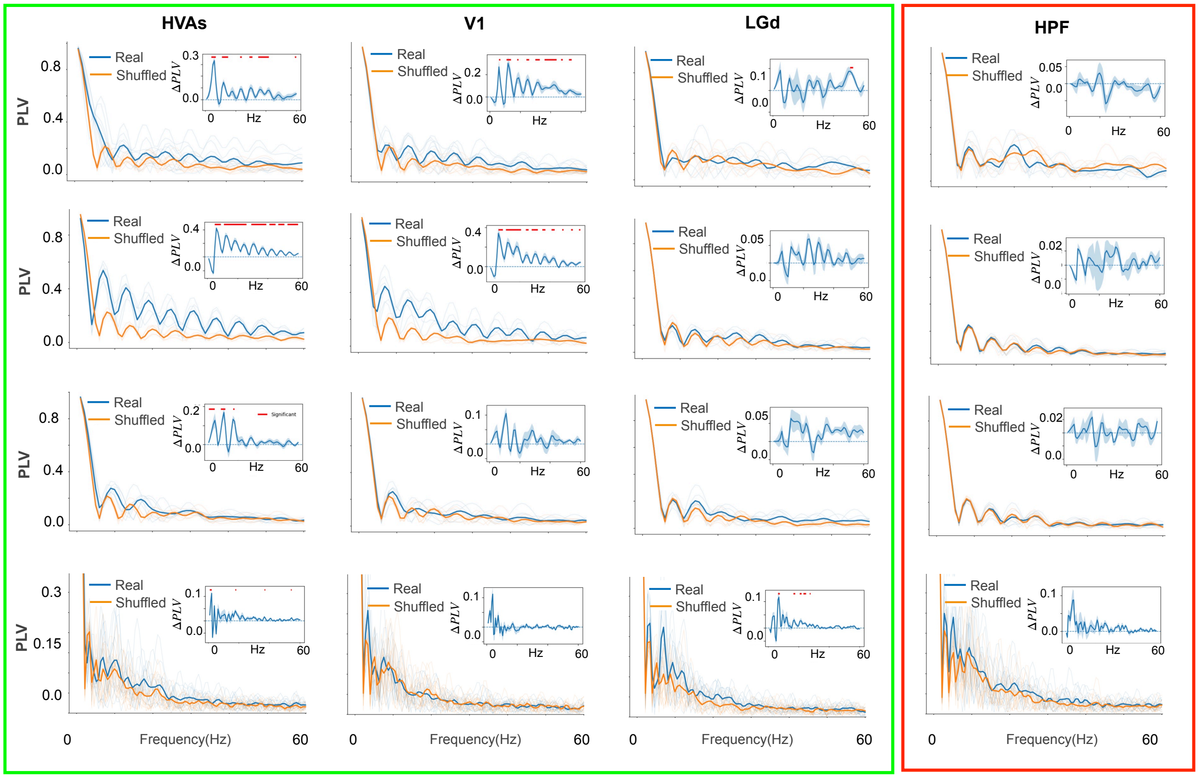

Supplementary Figure S4: **Persistence of bracket coding beyond the stimulus-onset response.** To assess the contribution of the first bracket boundary, marking the arrival of stimulus-evoked response in each region at about 50-70ms post stimulus onset, we repeated the PLV analysis after excluding troughs occurring within the first 70ms of each trial duration. Resulting PLV spectra are shown for real data (blue) and temporally shuffled controls (orange) across tasks and regions. Details parallel those in Figure 2 in the main text. As expected, removing the dominant early bracket reduced the overall PLV magnitude across visual regions. However, real PLVs remained higher than shuffled PLVs, particularly in HVAs and V1, indicating that bracket structure persists beyond the earliest stimulus-onset response. LGd showed a weaker real–shuffle separation that did not consistently reach significance, which is consistent with its lower bracket-coding strength in the visual hierarchy (Figure 4a).

bioRxiv preprint doi: https://doi.org/10.64898/2026.05.31.729124; this version posted July 22, 2026. The copyright holder for this preprint (which was not certified by peer review) is the author/funder, who has granted bioRxiv a license to display the preprint in perpetuity. It is made available under a CC-BY 4.0 International license.

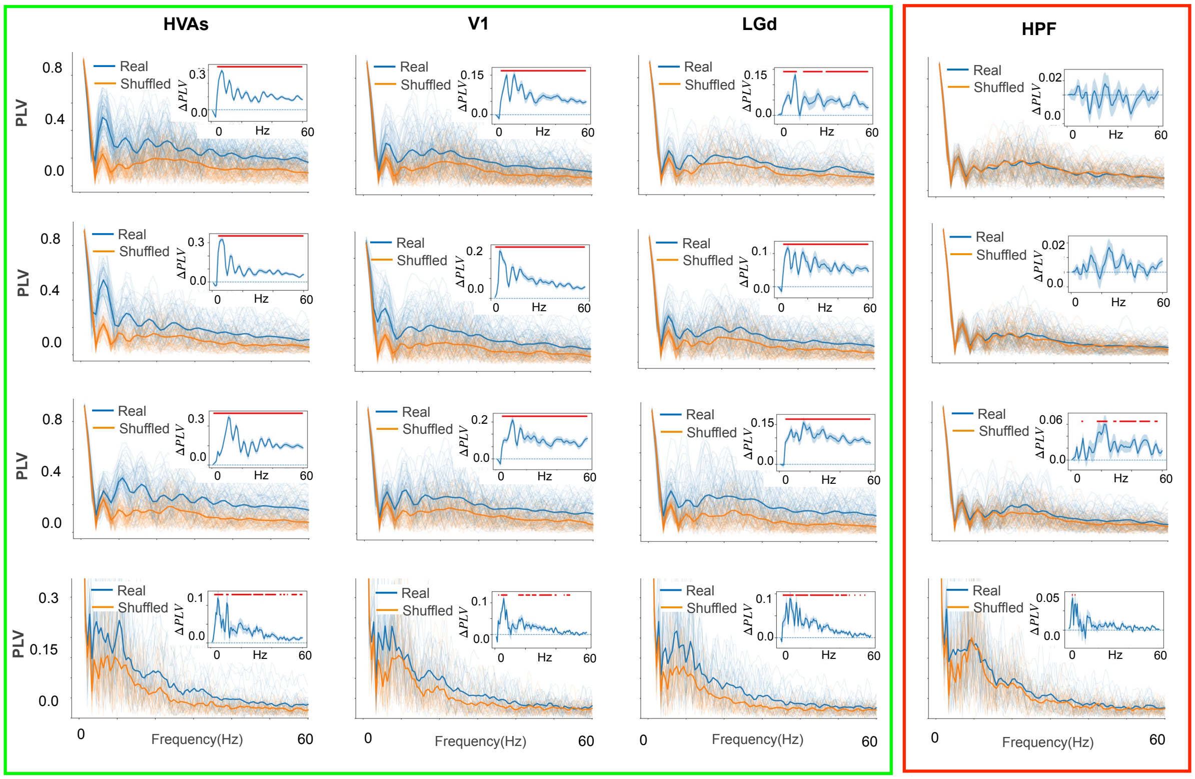

Supplementary Figure S5: **Subtask-specific temporal structure.** Complementary to the aggregated-trough PLV analyses in the main text (cf. trough rasters in Figure 2), we also computed PLVs for each subtask and then averaged the results, shown here for real data (blue) and temporally shuffled controls (orange). Details parallel those in Figure 2. As expected, mean subtask-level PLVs are generally higher than aggregated-trough counterparts. However, notably, the former also showed—almost exclusively in visual regions—stronger and more significant separation between real PLVs and shuffled surrogates, compared to the same separation in trough-aggregated counterparts. This result supports the interpretation that the elevated subtask-level PLVs in real data reflects temporal structure beyond what can be explained by firing rates alone.
